# *Rice black streaked dwarf virus* P7-2 forms a SCF complex through binding to *Oryza sativa* SKP1-like proteins, and interacts with GID2 involved in the gibberellin pathway

**DOI:** 10.1371/journal.pone.0177518

**Published:** 2017-05-11

**Authors:** Tao Tao, Cui-Ji Zhou, Qian Wang, Xiang-Ru Chen, Qian Sun, Tian-Yu Zhao, Jian-Chun Ye, Ying Wang, Zong-Ying Zhang, Yong-Liang Zhang, Ze-Jian Guo, Xian-Bing Wang, Da-Wei Li, Jia-Lin Yu, Cheng-Gui Han

**Affiliations:** 1 State Key Laboratory for Agro-biotechnology and Ministry of Agriculture Key Laboratory for Plant Pathology, China Agricultural University, Beijing, People's Republic of China; 2 Key Laboratory for Tobacco Gene Resources, Tobacco Research Institute, Chinese Academy of Agricultural Sciences, Qingdao, Shandong Province, People's Republic of China; National University of Singapore, SINGAPORE

## Abstract

As a core subunit of the SCF complex that promotes protein degradation through the 26S proteasome, S-phase kinase-associated protein 1 (SKP1) plays important roles in multiple cellular processes in eukaryotes, including gibberellin (GA), jasmonate, ethylene, auxin and light responses. P7-2 encoded by *Rice black streaked dwarf virus* (RBSDV), a devastating viral pathogen that causes severe symptoms in infected plants, interacts with SKP1 from different plants. However, whether RBSDV P7-2 forms a SCF complex and targets host proteins is poorly understood. In this study, we conducted yeast two-hybrid assays to further explore the interactions between P7-2 and 25 type I *Oryza sativa* SKP1-like (OSK) proteins, and found that P7-2 interacted with eight OSK members with different binding affinity. Co-immunoprecipitation assay further confirmed the interaction of P7-2 with OSK1, OSK5 and OSK20. It was also shown that P7-2, together with OSK1 and *O*. *sativa* Cullin-1, was able to form the SCF complex. Moreover, yeast two-hybrid assays revealed that P7-2 interacted with gibberellin insensitive dwarf2 (GID2) from rice and maize plants, which is essential for regulating the GA signaling pathway. It was further demonstrated that the N-terminal region of P7-2 was necessary for the interaction with GID2. Overall, these results indicated that P7-2 functioned as a component of the SCF complex in rice, and interaction of P7-2 with GID2 implied possible roles of the GA signaling pathway during RBSDV infection.

## Introduction

The S-phase kinase-associated protein 1 (SKP1) is a core component of the SKP1/Cullin/F-box (SCF) complex, an E3 ubiquitin ligase that triggers protein degradation through the 26S proteasome [1,2]. Within the SCF complexes, the scaffold-like Cullin binds to Rbx1 (Ring box1) to forms a core catalytic complex [3,4], the numerous F-box proteins bind the target protein and interact with SKP1 through their F-box motif [5,6], and SKP1 connects the highly variable F-box protein to Cullin [5,7]. There are two types of SKP1 proteins. Type I proteins contain two conserved domains (Skp1_POZ and Skp1) and two variable regions. Type II proteins are quite similar to type I proteins, but have an additional C-terminal region [8]. The *Arabidopsis thaliana* genome contains 21 *SKP1-like* genes (*ASK*s), of which 19 are type I and two are type II genes (*ASK20* and *ASK21*). In rice, there are at least 32 *Oryza sativa SKP1-like* genes (*OSK*s), of which 28 are type I [8]. SKP1 plays vital roles in gibberellin (GA), auxin, jasmonate (JA), ethylene and light responses through interaction with various F-box proteins to mediate degradation of different substrates [9–13], and many other cellular processes in eukaryotes [14]. Plant viruses were observed to hijack the components of SCF complexes to promote degradation of important cellular proteins and thus counter plant defense [15–17].

*Rice black streaked dwarf virus* (RBSDV), a member of the genus *Fijivirus* within the family of *Reoviridae*, is transmitted to maize, rice, wheat and barley via the small brown planthopper (*Laodelphax striatellus*) in a persistent, propagative manner [18,19]. RBSDV is a destructive viral pathogen that causes rice black streaked dwarf and maize rough dwarf diseases [20–22]. Plants infected with RBSDV show severe symptoms–obvious stunting, darkening of leaves and waxy galls along the veins [22,23]. The RBSDV virion is a double-layered, icosahedral particle that contains 10 segments of double-stranded RNA (S1–S10) [24,25]. Segments S1–S4, S6, S8 and S10 each have one open reading frame (ORF), encoding the RNA-dependent RNA polymerase (P1), core protein (P2), putative capping enzyme (P3), outer-shell B-spike protein of the virion (P4), viroplasm protein (P6), minor core capsid protein (P8) and the main outer capsid protein (P10) [24,26–28]. Each of the segments S5, S7 and S9 encodes two proteins. P5-1 is a putative component of viroplasms, where virus replication occurs [29]. P7-1 participates in forming the tubular structures [28,30] and P9-1 is a main viroplasm matrix protein [31–33]. The functions of P5-2, P7-2 and P9-2 are poorly understood.

RBSDV P7-2 encoded by ORF2 in segment S7, is a nonstructural protein containing 309 amino acids [28]. Detection of P7-2 in RBSDV-infected plants or *L*. *striatellus* has failed using antiserum against P7-2 [28], probably resulting from a low translation efficiency of the dicistronic mRNA [34]. Indeed, the mRNA level of P7-2 from *Southern rice black-streaked dwarf virus*, a close relative of RBSDV, is unusually low in different hosts as detected using quantitative real-time PCR [35]. A previous study showed that P7-2 interacts with *Zea mays* SKP1 of SCF complex, suggesting its involvement in plant-virus interaction through the ubiquitylation pathway [36]. It was further found that P7-2 also binds to SKP1 proteins from *Nicotiana benthamiana*, *A*. *thaliana*, *O*. *sativa* and *Saccharum sinense*, all of which belong to the SKP1 type I proteins [36]. However, it is not known whether P7-2 forms a SCF complex through interaction with SKP1 and thus triggers degradation of cellular proteins.

Phytohormones including salicylic acid, JA and ethylene, play crucial roles in activating the plant innate immune system and modulating defense against invading pathogens through regulatory crosstalk between signaling pathways [37–39]. Other plant hormones, including GAs, abscisic acid, auxin, cytokinins and brassinosteroids (BRs) are also vital in regulating plant-microbe interactions [40,41].

The GAs are tetracyclic diterpenoid phytohormones that are necessary for various aspects of plant growth and development, including seed germination, stem elongation, leaf expansion, trichome development, pollen maturation and floral transition [42]. GA 20-oxidases and GA 3-oxidases are GA biosynthetic enzymes that control the synthesis of two main bioactive GAs (GA_4_ and GA_1_) via a series of oxidation steps [43]. The GA receptor gibberellin insensitive dwarf1 (GID1), the nuclear DELLA growth repressing proteins (DELLAs), the F-box proteins SLEEPY1 (SLY1) and SNEEZY (SNZ) in *A*. *thaliana* and gibberellin insensitive dwarf2 (GID2) in rice are key players involved in the GA signaling pathway [44]. GA perception is mediated by a soluble receptor, GID1 [45]. Interaction of bioactive GA with GID1 triggers a conformational change of GID1, which allows the nuclear growth inhibitors DELLAs to bind to GID1 by its DELLA/TVHYNP domains [46–48]. The GA–GID1–DELLA complex facilitates the binding of DELLA to the E3 ligase SCF^SLY1/GID2^, promoting the ubiquitylation and subsequent destruction of DELLA through the 26S proteasome [13,49–51]. Thus, GA facilitates plant growth by controlling the degradation of DELLA proteins in a proteasome-dependent manner. It has been shown that the F-box protein GID2 interacts with OSK1, OSK13, OSK20 and OSK25 [13,52], and acts as a component of the SCF complex in rice [13]. In the GA signaling pathway, GID2 acts as a positive regulator that interacts with the phosphorylated DELLA protein Slender Rice 1 (SLR1) and triggers degradation of SLR1 through the ubiquitin/proteasome pathway [13]. Mutant plants lacking GA show a dwarf phenotype [51]. RBSDV causes dwarfed growth abnormality in infected rice and maize plants. It is reported that concentration of GA_3_ is lower in RBSDV-infected plants than in healthy plants [53]. Participation of plant hormones JA and BR in RBSDV infection was recently described [54]; however, the role that GA plays during RBSDV infection remains to be elucidated.

It is not known whether RBSDV P7-2 forms a SCF complex. In this study, we further explored the interactions between P7-2 and 25 type I OSKs. We found that P7-2 could bind eight OSK members using yeast two-hybrid assays, and interactions of P7-2 with OSK1, OSK5 and OSK20 were further confirmed by co-immunoprecipitation (co-IP). A yeast three-hybrid assay showed that P7-2, OSK1 and *O*. *sativa* Cullin-1 (OsCUL1) were able to form the SCF complex. Moreover, yeast two-hybrid assays revealed that P7-2 interacted with GID2 from rice and maize plants. Further study showed that the N-terminal region of P7-2 was responsible for the interaction with GID2.

## Materials and methods

### Plant materials and growth conditions

The *N*. *benthamiana* plants used in this study were grown and maintained at 24°C with 16 h light and 8 h darkness.

### Yeast two-hybrid assay

The *P7-2* was amplified from plasmid pHbm-S7 containing the full-length cDNA of RBSDV S7 (AF397894) as previously described [36]. The *P7-2*, *P7-2*^*1–287*^, *P7-2*^*1–295*^, *P7-2*^*25–309*^, *P7-2*^*44–309*^ and *P7-2*^*79–309*^ constructs were cloned into pGBKT7 vector (Clontech, Mountain View, CA, USA) as described previously [36] and were transformed into yeast strain Y187. *OSK1* (Os11g26910), *OSK2* (Os10g30200), *OSK3* (Os02g13180), *OSK4* (Os09g10200), *OSK5* (Os09g10260), *OSK6* (Os07g05180), *OSK7* (Os09g10300), *OSK9* (Os07g05150), *OSK10* (Os06g02360), *OSK11* (Os06g02350), *OSK12* (Os09g10270), *OSK13* (Os09g10230), *OSK14* (Os09g0272900), *OSK15* (Os08g28820), *OSK16* (Os07g05160), *OSK17* (Os07g43180), *OSK19* (Os07g43260), *OSK20* (Os09g36830), *OSK21* (Os07g22680), *OSK22* (Os07g43250), *OSK25* (Os08g28800), *OSK26* (Os07g43220), *OSK27* (Os07g43230), *OSK28* (Os07g43240), *OSK29* (Os08g28780), *O*. *sativa GID1* (*OsGID1*, AB211399), *O*. *sativa GID2* (*OsGID2*, AB100246), *O*. *sativa SLR1* (*OsSLR1*, AB262980) and *Z*. *mays GID2* (*ZeaGID2*, NM_001155936) were cloned by reverse transcription-PCR (RT-PCR) method and inserted into pGADT7 vector (Clontech), the resulting constructs were transformed into yeast strain AH109. All primers used for construction of recombinant plasmids mentioned above are listed in S1 Table. Yeast two-hybrid analyses were conducted using the Matchmaker GAL4 Two-Hybrid System 3 (Clontech) as described previously [55]. Co-transformants were selected on synthetic dropout (SD) media lacking leucine and tryptophan (SD/-Leu/-Trp) and grown at 30°C for 48–72 h. Interactions between two proteins were validated by growth on SD media lacking leucine, tryptophan, adenine and histidine (SD/-Leu/-Trp/-Ade/-His) and containing 5-bromo-4-chloro-3-indolyl−β-D-galactoside (X-α-gal). For serial dilution analysis, exponentially grown yeast cells were collected and adjusted to OD_600_ = 1.0 and diluted to 10^−1^, 10^−2^ and 10^−3^.

### *In vivo* co-IP

*P7-2* was inserted into the vector pMDC32 [56] containing 3xFlag at the C-terminus, and *GFP* was introduced into the vector pGD-3xFlag, a modified binary vector pGD with 3xFlag at its C-terminus [57]. *OSK1*, *OSK5*, *OSK20* and *GFP* were cloned into pGD-6xMyc that contained 6xMyc tag at the C-terminus [58]. Primers used for constructing the recombinant plasmids mentioned above are shown in S1 Table. The *35S*:*P7-2*-*3xFlag* construct was transiently expressed with *35S*:*OSK1*-*6xMyc*, *35S*:*OSK5*-*6xMyc* and *35S*:*OSK20*-*6xMyc*, respectively, together with *35S*:*P19* [59] in *N*. *benthamiana* leaves by *Agrobacterium*-mediated infiltration method as described previously [55]. GFP-6xMyc and GFP-3xFlag served as negative controls. *In vivo* co-IP was performed [60]. Briefly, 72 h after infiltration, 3 g of the infiltrated leaves were collected and ground in liquid nitrogen, suspended in an equal volume (w/v) of extraction buffer (150 mM NaCl, 25 mM Tris-HCl, pH 7.5, 10 mM Dithiothreitol, 1 mM Ethylenediaminetetraacetic acid (EDTA), 10% glycerol, 2% w/v polyvinylpolypyrrolidone, 1 × proteinase inhibitor cocktail and 0.1% Triton-X 100). After vigorous vortexing, the suspension was placed on ice for 30–60 min. A 200-mesh nylon net was used to filtrate the suspension to exclude the cell walls and other impurities and then centrifuged at 12,000 rpm for 15 min. The resulting supernatant was homogenized on a mute mixer at 4°C for 4 h with anti-Flag beads that had been balanced in IP buffer (150 mM NaCl, 25 mM Tris-HCl, pH 7.5, 1 mM EDTA, 10% glycerol and 0.1% Triton-X 100) and blocked with Bovine serum albumin. The precipitated samples were extensively washed nine times with IP buffer, and then boiled with 2 × sodium dodecyl sulfate (SDS) sample buffer (100 mM Tris-HCl, pH 6.8, 20% glycerol, 4% SDS, 0.2% bromophenol blue and 5% β-mercaptoethanol added before use) for 10 min.

### Sodium dodecyl sulfate-polyacrylamide gel electrophoresis (SDS-PAGE) and western blot analysis

SDS-PAGE and western blot analysis were conducted as described [36,55]. Briefly, samples prepared from co-IP were separated by 12.5% SDS polyacrylamide gel electrophoresis (PAGE) and western blot analysis was performed by adding an anti-Flag polyclonal antibody (1:5000; Abmart, Berkeley Heights, NJ, USA) or an anti-c-Myc polyclonal antibody (1:5000; Abmart) followed by a goat anti-mouse horseradish peroxidase secondary polyclonal antibody (1:3000; Bio-Rad, Hercules, CA, USA) or an goat anti-rabbit horseradish peroxidase secondary polyclonal antibody (1:3000; Bio-Rad). Signals were detected with an enhanced chemiluminescence detection kit (GE Healthcare, Buckinghamshire, UK) according to the manufacturer’s instructions.

### Yeast three-hybrid assay

The *P7-2* and *OSK1*/*OSK5*/*OSK20* were cloned into the pBridge vector (Clontech) to produce fusions with the GAL4 DNA-binding domain (BD) and Met promoter, respectively, and were transformed into yeast strain Y187. *OsCUL1* was inserted into the pGADT7 vector (Clontech) to generate pGAD-OsCUL1 and was transformed into yeast strain AH109. Double transformants were selected on dropout meida (SD/-Met/-Leu/-Trp). Protein interactions were confirmed by growth on selective media (SD/-Met/-Leu/-Trp/-His/-Ade, X-α-gal). Serial dilution analysis was performed as described previously. S1 Table shows the primers used for construction of the aforementioned recombinant plasmids.

## Results

### P7-2 interacted with eight OSK proteins in yeast two-hybrid assays

**Fig 1:**
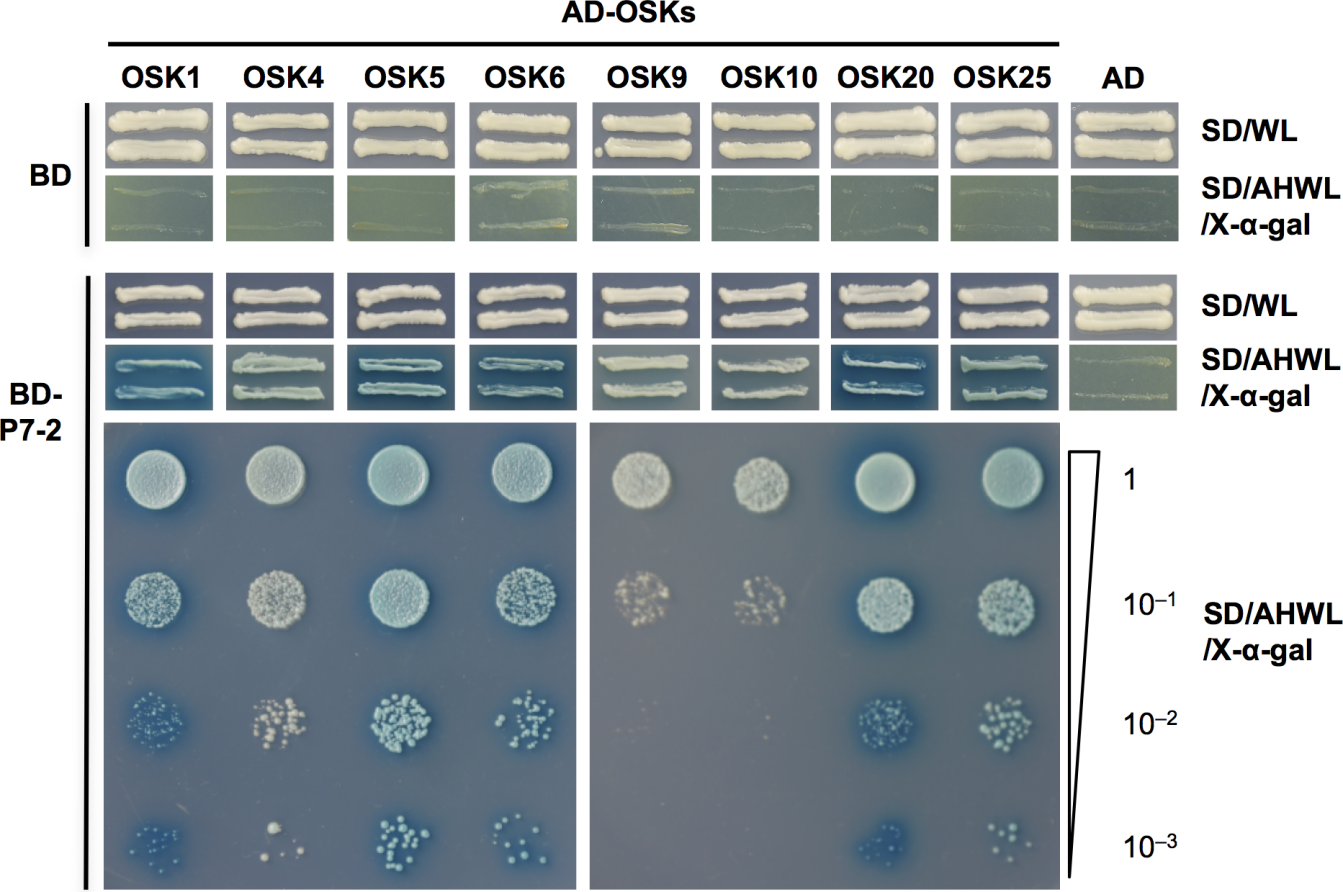
Determination of the interactions between P7-2 and rice OSK proteins by yeast two-hybrid assay. The growth of yeast cells harboring the P7-2 fused to the GAL4 DNA-binding domain (BD), and OSK proteins fused to the GAL4 activation domain (AD) are shown. SD/WL was the nonselective medium and SD/AHWL/X-α-gal was the selective medium. For serial dilution analysis, yeast cells were collected and adjusted to OD_600_ = 1.0, and diluted to 1, 10^−1^, 10^−2^ and 10^−3^. Corresponding yeast transformants were spotted on SD/AHWL/X-α-gal. Plasmids expressing only the DNA-activation domain or DNA-binding domain were used as negative controls. SD/WL, -Trp-Leu; SD/AHWL/X-α-gal, -Ade-His-Trp-Leu containing X-α-gal.

It has been reported that P7-2 interacts with OSK1 protein [36], which is a type I SKP1. To determine whether P7-2 could bind other type I OSK proteins, total RNA was extracted from 4-week-old rice plants (*O*. *sativa* L. *japonica* cv. Nipponbare), and 25 out of 28 type I *OSK* genes were cloned by RT-PCR: *OSK1*–*OSK7*, *OSK9*–*OSK17*, *OSK19*–*OSK22* and *OSK25*–*OSK29*. To examine the interactions between P7-2 and other type I OSKs, we subjected the obtained 25 OSKs to yeast two-hybrid analysis with P7-2. Briefly, P7-2 was fused to the GAL4 DNA-BD, and the 25 OSKs were fused to the GAL4 activation domain (AD), respectively. The resulting plasmids BD-P7-2 and AD-OSKs (OSK1–OSK7, OSK9–OSK17, OSK19–OSK22 and OSK25–OSK29) were transformed into yeast strains Y187 and AH109, respectively, to implement a yeast two-hybrid mating strategy. Mixture of the Y187 and AH109 transformants grew vigorously on SD/-Leu/-Trp medium (Fig 1). However, only the diploid yeast strains produced from mating of BD-P7-2 in Y187 and AD-OSKs (OSK1, OSK4, OSK5, OSK6, OSK9, OSK10, OSK20 and OSK25) in AH109, respectively, were able to grow on selective media (SD/-Leu/-Trp/-Ade/-His) that contained X-α-gal (Fig 1). The double transformants expressing BD-P7-2 and AD-OSKs (OSK1, OSK5, OSK6, OSK20 and OSK25) grew on the selective media and turned blue (Fig 1). The results showed that P7-2 had the ability to bind OSK1, OSK4, OSK5, OSK6, OSK9, OSK10, OSK20 and OSK25 with different binding capabilities in yeast (Fig 1). Specifically, P7-2 interacted strongly with OSK1, OSK5, OSK6, OSK20 and OSK25, and could bind OSK4, OSK9 and OSK10 with low affinity (Fig 1).

### Interactions of P7-2 with OSK1, OSK5 and OSK20 confirmed by *in vivo* co-IP

**Fig 2:**
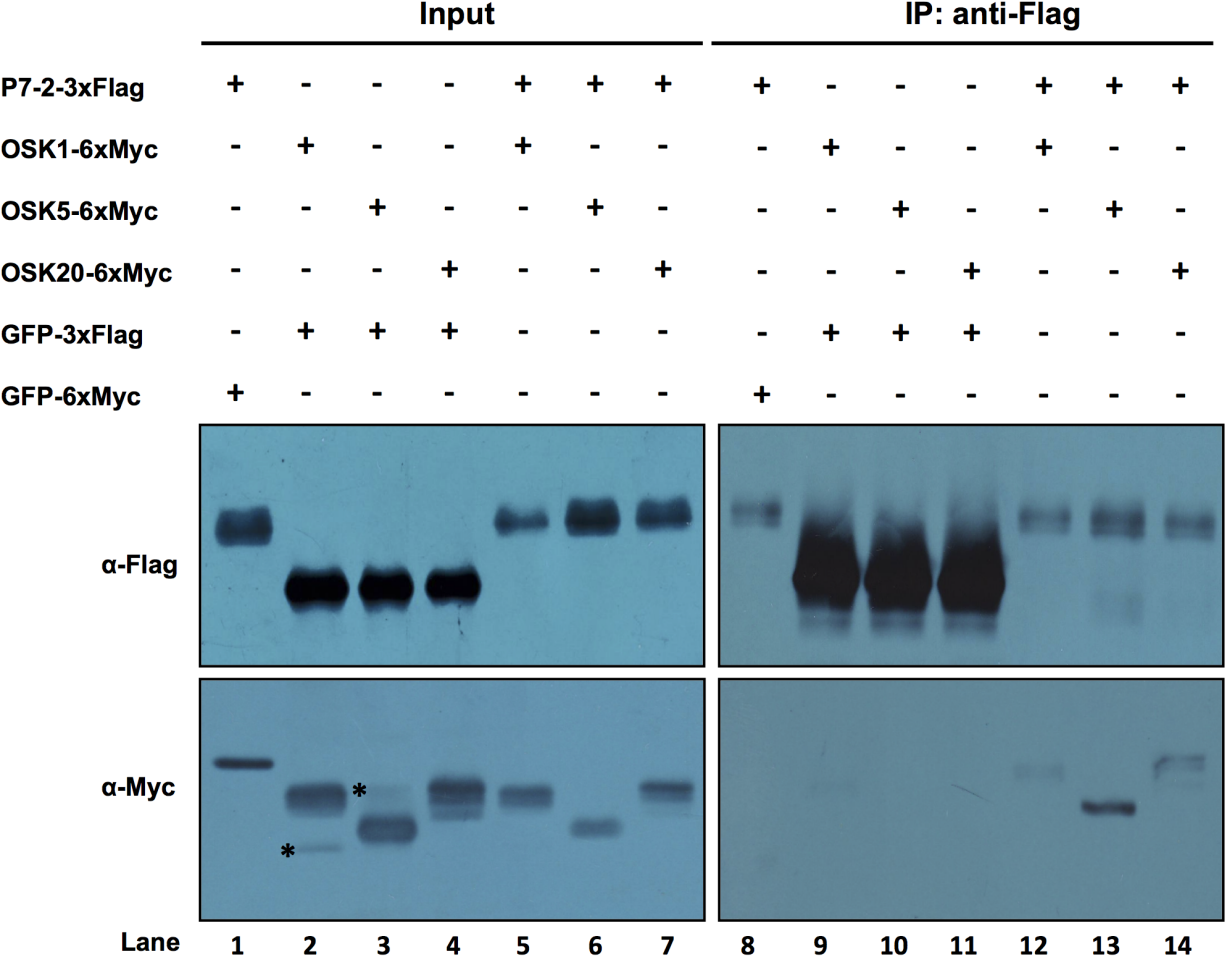
Co-immunoprecipitation of P7-2-3xFlag and OSK1-6xMyc/OSK5-6xMyc/OSK20-6xMyc from infiltrated *Nicotiana benthamiana* leaves. Total protein extracts (Input) or immunoprecipitated (IP) fractions using an anti-Flag antibody were analyzed by immunoblotting using anti-Flag or anti-Myc antibodies. GFP-3xFlag and GFP-6xMyc were used as negative controls. The asterisks indicate non-specific signals.

As P7-2 interacted most strongly with OSK1, OSK5 and OSK20 in yeast (Fig 1), a co-IP assay was conducted to further verify these interactions. The *35S*:*P7-2-3xFlag* construct was transiently expressed with *35S*:*OSK1-6xMyc*, *35S*:*OSK5-6xMyc*, or *35S*:*OSK20-6xMyc*, respectively, in *N*. *benthamiana* leaves through *Agrobacterium*-mediated infiltration method. The *35S*:*GFP-6xMyc* and *35S*:*GFP-3xFlag* were used as negative controls. At 72 h after infiltration, total leaf proteins were extracted from inoculated leaves and were immunoprecipitated with anti-Flag beads. The bound proteins were detected by western blot with anti-Flag and anti-Myc antibodies. The results showed that OSK1-6xMyc, OSK5-6xMyc and OSK20-6xMyc were respectively co-immunoprecipitated with P7-2-3xFlag by anti-Flag antibody (Fig 2). No signal was detected for the negative controls (GFP-3xFlag and GFP-6xMyc) (Fig 2). Thus, P7-2 interacted with OSK1, OSK5 and OSK20 *in vivo*.

### P7-2, OSK1 and OsCUL1 formed SCF complex in yeast

**Fig 3:**
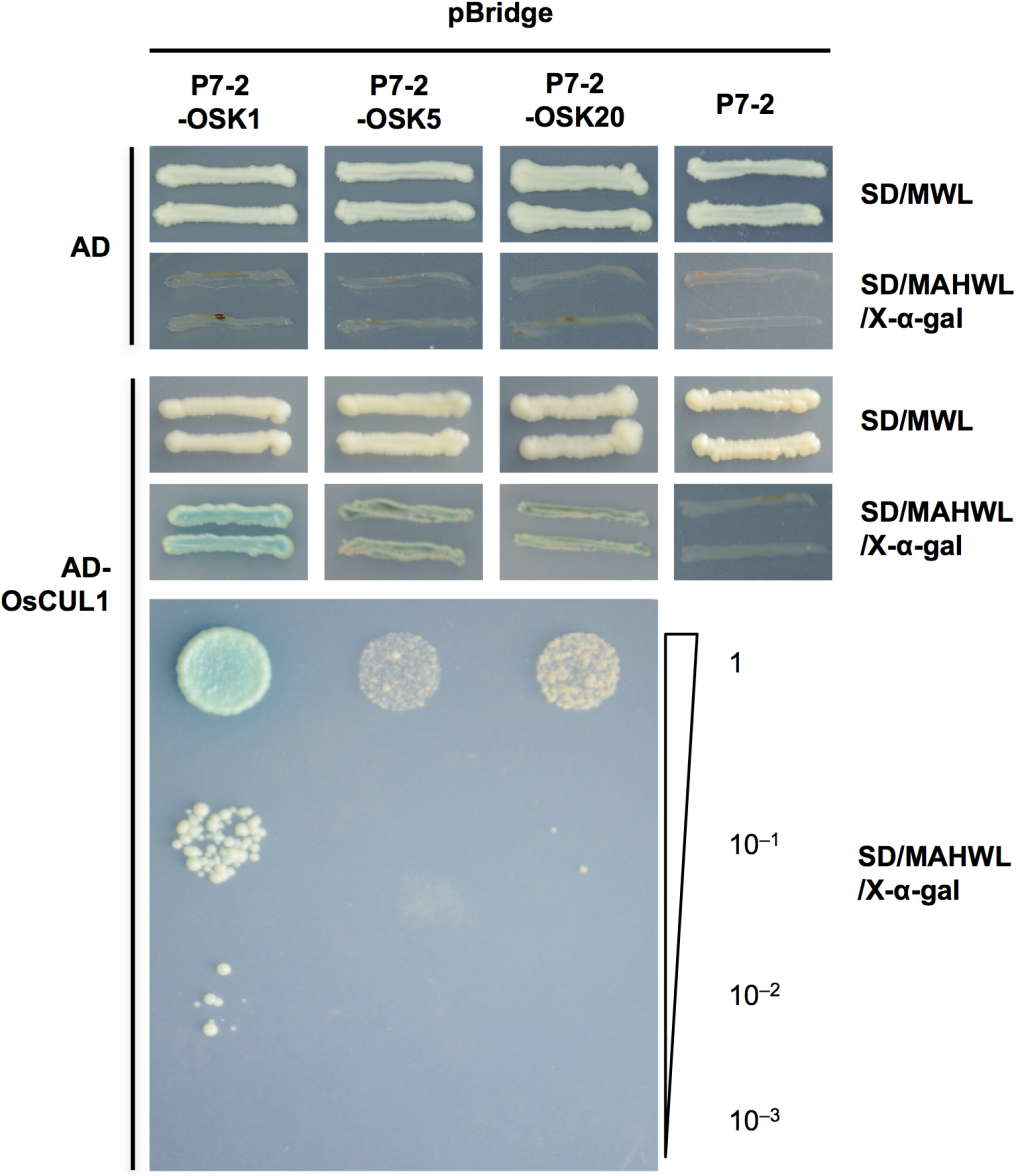
Yeast three-hybrid assay to identify the interaction of P7-2, OSKs (1, 5 and 20) and OsCUL1. The growth of yeast cells containing P7-2 fused to the GAL4 DNA-binding domain (BD), OSK1/OSK5/OSK20 fused to the Met promoter, and OsCUL1 fused to the GAL4 activation domain (AD) are shown. SD/MWL was the nonselective medium and SD/MAHWL/X-α-gal was the selective medium. Media lacking Met were used to induce the expression of the OSKs (1, 5 and 20) protein by Met promoter for testing the interaction between the three proteins expressed. For serial dilution analysis, collected yeast cells were adjusted to OD_600_ = 1.0, and diluted to 1, 10^−1^, 10^−2^ and 10^−3^. pGADT7 vector expressing only the DNA activation domain or pBridge vector expressing P7-2 alone served as negative controls. SD/MWL, -Met-Trp-Leu; SD/MAHWL/X-α-gal, -Met-Ade-His-Trp-Leu containing X-α-gal.

The yeast two-hybrid and co-IP assays revealed that P7-2 had the ability to bind OSKs. To explore whether P7-2 functioned as a subunit of the SCF complex, we tested the interactions of P7-2, OSKs (1, 5 and 20) and OsCUL1 by yeast three-hybrid assay. The yeast strains expressing P7-2, OSK1 and OsCUL1 grew well on the selective medium (SD/-Met/-Leu/-Trp/-His/-Ade, X-α-gal) and turned blue (Fig 3), showing positive interactions among P7-2, OSK1 and OsCUL1. The yeast strains containing P7-2, OSK5/OSK20 and OsCUL1 grew on the same selective medium but did not turn blue (Fig 3), indicating a weak interaction among P7-2, OSKs (5 and 20) and OsCUL1. No interaction was observed between P7-2 and OsCUL1 in the absence of OSKs (1, 5 and 20) (Fig 3), indicating that OSKs (1, 5 and 20) were necessary for interaction between P7-2 and OsCUL1. The results revealed that P7-2, OSK1 and OsCUL1 formed a ternary complex in yeast, but interactions among P7-2, OSK5/OSK20 and OsCUL1 were relatively weak.

### P7-2 interacted with GID2 from rice and maize plants

**Fig 4:**
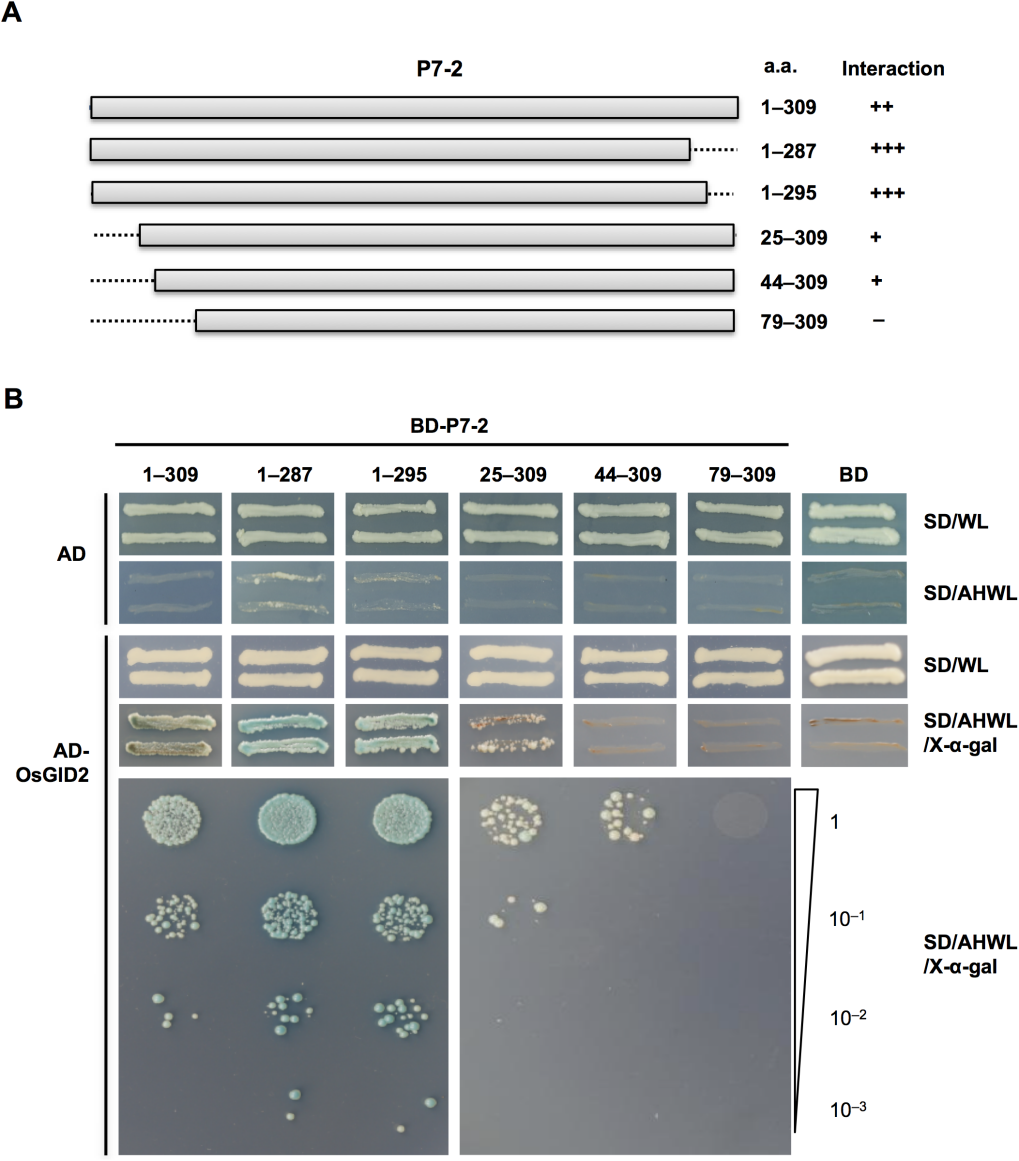
Interaction between P7-2 and OsGID2. (A) Schematic of P7-2 and the truncations used is shown. Bars indicate the full-length P7-2 (a.a. 1–309) and the truncations, and dashed lines show the deleted regions. The binding affinity between P7-2 truncations and OsGID2 is shown (+, positive;–, negative). a.a., amino acid. (B) Analysis of the regions of P7-2 responsible for binding to OsGID2 by yeast two-hybrid assay. The growth of yeast transformants containing different truncated forms of P7-2 fused to BD, and OsGID2 fused to AD are shown. SD/WL was the nonselective medium, and the interactions were confirmed by the growth of yeast cells on the SD/AHWL/X-α-gal. For serial dilution analysis, yeast cells were collected and adjusted to OD_600_ = 1.0, and diluted to 1, 10^−1^, 10^−2^ and 10^−3^. Plasmids expressing only the DNA-activation domain or DNA-binding domain were used as negative controls. Numbers indicate a.a. residues of P7-2 fused to BD. SD/WL, -Trp-Leu; SD/AHWL/X-α-gal, -Ade-His-Trp-Leu containing X-α-gal.

**Fig 5:**
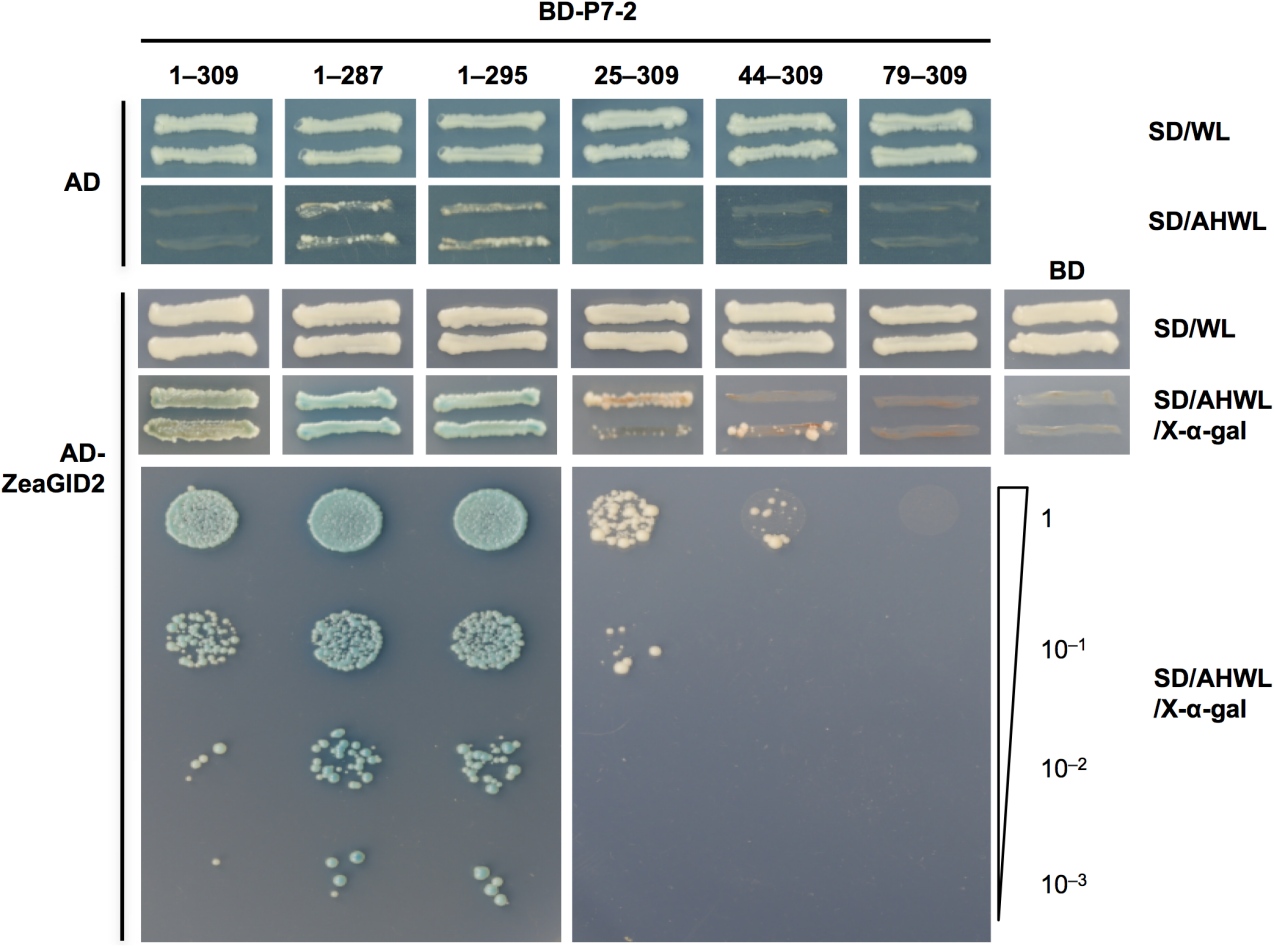
Determination of regions of P7-2 necessary for binding to ZeaGID2 by yeast two-hybrid assay. SD/WL was the nonselective medium, and the interactions were verified by the growth of yeast transformants on SD/AHWL/X-α-gal. Numbers show the different truncations of P7-2 fused to BD. To conduct serial dilution analysis, yeast cells were collected and adjusted to OD_600_ = 1.0, and diluted to 1, 10^−1^, 10^−2^ and 10^−3^. Plasmids expressing only the DNA-activation domain or DNA-binding domain were used as negative controls. SD/WL, -Trp-Leu; SD/AHWL/X-α-gal, -Ade-His-Trp-Leu containing X-α-gal.

It has been reported that RBSDV caused stunting in rice and maize plants, and a decrease of GA_3_ contents was observed in RBSDV-infected plants [53]. To test whether P7-2 interacted with OsGID1, OsSLR1 and OsGID2, which are key proteins involved in GA signaling pathway [44], a yeast two-hybrid assay was conducted. Total RNA was extracted from 4-week-old rice plants, and *OsGID1*, *OsGID2* and *OsSLR1* were cloned by RT-PCR and inserted into pGADT7 to gain AD-OsGID1, AD-OsGID2 and AD-OsSLR1, respectively. We found that P7-2 interacted with OsGID2 (Fig 4A and 4B), but did not bind OsGID1 and OsSLR1 in yeast (S1 Fig). GID2 from maize plants was also cloned by RT-PCR and introduced into pGADT7 to test the interaction between P7-2 and ZeaGID2. The yeast two-hybrid assay showed that P7-2 also interacted with ZeaGID2 protein (Fig 5).

### N-terminal region of P7-2 was necessary for interaction between P7-2 and OsGID2/ZeaGID2

We performed yeast two-hybrid assays to identify the key region of P7-2 that interacted with GID2. Several P7-2 truncation derivatives expressing BD-P7-2^1–287^, BD-P7-2^1–295^, BD-P7-2^25–309^, BD-P7-2^44–309^ and BD-P7-2^79–309^ were constructed (Fig 4A). The yeast cells expressing the C-terminal truncated mutants BD-P7-2^1–287^ or BD-P7-2^1–295^ together with AD-OsGID2 grew on the selective medium (SD/-Met/-Leu/-Trp/-His/-Ade, X-α-gal) and turned blue (Fig 4B). We also found that OsGID2 had higher binding affinity towards BD-P7-2^1–287^ and BD-P7-2^1–295^, compared with intact P7-2 (Fig 4B). However, the N-terminal deleted mutants BD-P7-2^25–309^ and BD-P7-2^44–309^ had weakened interaction between P7-2 and OsGID2 compared with full-length P7-2 (Fig 4B). When amino acids 1–78 of P7-2 were deleted, interaction between P7-2 and OsGID2 was abolished (Fig 4B). Similar results were observed for ZeaGID2 protein (Fig 5). These results demonstrated that the N-terminal region of P7-2 was essential for its binding to OsGID2 and ZeaGID2.

## Discussion

In this study, we investigated the interaction between P7-2 and 25 type I OSKs using yeast two-hybrid assays. The results showed that P7-2 interacted with eight OSKs with different binding affinity (Fig 1). Co-IP further validated the interactions of P7-2 with OSK1, OSK5 and OSK20 (Fig 2). It has been demonstrated that some F-box proteins can bind one or more *Arabidopsis* ASK proteins [61]. Wang reported that P7-2 interacted with OSK1 [36]. Our study showed that P7-2 not only interacted with OSK1, but could also bind OSK4, OSK5, OSK6, OSK9, OSK10, OSK20 and OSK25 (Fig 1). Binding to P7-2 may render OSKs inaccessible for other host proteins and thus disturb some physiological processes in plants. It was previously found that the 21 *Arabidopsis* ASK proteins exhibit considerable differences in binding capabilities to to various F-box proteins [62]. Here, we found that varied OSKs interacted with P7-2 with different binding abilities (Fig 1). P7-2 interacted strongly with OSK1, OSK5, OSK6, OSK20 and OSK25, but bound OSK4, OSK9 and OSK10 with low affinity (Fig 1). Sequence alignment of OSK proteins interacting with P7-2 indicated that different binding affinity among these OSKs towards P7-2 might arise from key amino acid changes (S2 Fig). There are 26 key amino-acids in the human SKP1 (Hs-SKP1) that have been reported to connect the human SKP2 F-box protein [7,63]. These amino acid residues closely related to the interaction between SKP1 and F-box proteins are also conserved among SKP1 proteins from plant species [52,64]. In terms of sequence alignment, four out of the 26 crucial amino-acids in OSK10 were changed compared with the OSKs that interacted strongly with P7-2 (S2 Fig). Specifically, the polar amino acids Q/N/K/R at site 142 (referring to OSK10) were changed to the nonpolar amino acid I, the amino acid C at site 165 was changed to A, the nonpolar amino acid F at site 190 was changed to the polar amino acid H, and the amino acid N at site 202 was changed to Y compared with the OSKs that showed high binding affinity to P7-2 (S2 Fig). Alterations in these four crucial amino acid residues might result in low binding affinity of OSK10 to P7-2. It was reported that Hs-SKP1 contains eight helices (H1–H8) [63], and these helices are also conserved among other SKP1 proteins [52]. Helices H5–H8 of Hs-SKP1 form the human SKP2 F-box protein-binding site [63,65]. The 106^th^ residue of OSK4 in H5 was changed from polar amino acids D/N/E to nonpolar amino acid G (S2 Fig). Similar to OSK4, the 98^th^ residue of OSK9 in H5 was changed from the polar amino acids D/N/E to the nonpolar amino acid G, and a Glu (E) residue of OSK9 in H8 was missing compared with the OSKs that interacted strongly with P7-2 (S2 Fig). These amino acid changes in H5 and/or H8 of OSK4 and OSK9 might contribute to their weak interaction with P7-2. The *OSK*s show different expression patterns in various growth stages of rice. *OSK1*, *OSK8*, *OSK11*, *OSK20* and *OSK23* are widely and strongly expressed [52], suggesting their involvement in diverse developmental processes. Particularly, *OSK1* is the most strongly and widely expressed *OSK* gene. However, most *OSK*s are expressed in flowers [52]. Kahlou *et al*. showed that OSK1 and OSK20 bind to a majority of the nine F-box proteins examined in a yeast two-hybrid assay [52], suggesting that OSK1 and OSK20 have a role in forming various SCF complexes. In accordance, P7-2 had a high affinity for OSK1 and OSK20 in yeast (Fig 1).

The SCF complex is an E3 ubiquitin ligase, mediating protein degradation through the 26S proteasome [1,2]. Here, we demonstrated that P7-2 associated with OSK1 and OsCUL1 to form a SCF complex (Fig 3), suggesting the involvement of P7-2 in ubiquitin-mediated degradation of cellular proteins in rice. Plant viruses were found to act as a F-box protein and interact with SKP1 to target important host factors [15]. For example, P0 proteins encoded by the poleroviruses *Cucurbit aphid-borne yellows virus* and *Beet western yellows virus* interact with *Arabidopsis* homologs of SKP1, *Arabidopsis* SKP1-like 1 (ASK1) and ASK2 through their F-box motif [17] to target ARGONAUTE1 (AGO1) [66,67], the core subunit of the RNA-induced silencing complex functioning in the RNA silencing pathway [68]. In addition, the F-box protein CLINK (Cell cycle link) from the nanovirus *Faba bean necrotic yellow virus* interacts with SKP1 and the retinoblastoma tumor-suppressor protein pRB. Through inactivation of pRB, the virus is able to affect cell cycling and thus facilitates its replication [16].

In the current study, yeast two-hybrid assays showed that P7-2 interacted with both OsGID2 and ZeaGID2 (Figs 4 and 5). In addition, we found that the N-terminal region of P7-2 was essential for the interaction with GID2 (Figs 4 and 5). GID2 is involved in the GA signaling pathway and functions as a component of the SCF complex that specifically interacts with the phosphorylated DELLA protein SLR1 and triggers the ubiquitin-mediated degradation of SLR1 in rice [13]. It is possible that binding of P7-2 to GID2 might impair the interaction between GID2 and SLR1 by hijacking GID2, which might result in increased accumulation of SLR1 protein in RBSDV-infected plants or plants overexpressing P7-2. It was reported that the F-box protein GID2 interacted with OSK1, OSK13, OSK20 and OSK25 [13,52]. Our results also implied that P7-2 might interfere with the interaction between GID2 and OSKs by hijacking GID2 and OSKs. The capacity of P7-2 to bind both GID2 and OSKs also offered the possibility that P7-2 promoted the ubiquitin-mediated degradation of GID2. It is reported that the P2 protein encoded by *Rice dwarf virus* interacts with ent-kaurene oxidases, which play an essential role in synthesis of GAs, leading to lower concentration of GA and dwarf symptoms in rice [69]. RBSDV infection causes dwarf symptoms in rice and maize plants, and the endogenous GA concentration is reduced in response to RBSDV infection [53]. However, whether P7-2 affects the accumulation of GID2 remains to be investigated. Participation of plant hormones in RBSDV infection was described recently. It was revealed that genes in the JA pathway were induced, whereas the genes involved in the BR pathway were down-regulated in RBSDV-infected rice plants. It was further demonstrated that JA suppresses RBSDV infection and BR mediates susceptibility to RBSDV infection through infection assay using JA-insensitive mutant *coi1-13* and BR-insensitive mutant *Go* [54]. However, the relationship between GA and RBSDV infection is poorly understood. Our results showed that P7-2 interacted with GID2, the crucial protein functioning in the GA signaling in rice, suggesting that GA might play a role in RBSDV infection.

In conclusion, our results demonstrated that RBSDV P7-2 interacted with eight OSKs by yeast two-hybrid assays, and the capacity of P7-2 to bind OSK1, OSK5 and OSK20 was further validated by co-IP. Yeast three-hybrid assay revealed that P7-2 associated with OSK1 and OsCUL1 to form the SCF complex. In addition, yeast two-hybrid assays showed that P7-2 interacted with OsGID2 and ZeaGID2 through its N-terminal region. These results demonstrated that RBSDV P7-2 served as a component of the SCF complex in rice, and suggested that the GA signaling pathway may play some role during RBSDV infection.

## Supporting information

S1 Fig P7-2 did not interact with OsGID1 and OsSLR1 as shown by yeast two-hybrid assay. SD/WL was the nonselective medium and SD/AHWL/X-α-gal was the selective medium. SKP1 from *Nicotiana benthamiana* (NbSKP1) fused to DNA-binding domain was served as positive control. SD/WL, -Trp-Leu; SD/AHWL/X-α-gal, -Ade-His-Trp-Leu containing X-α-gal.
(TIFF)

S2 Fig Sequence alignment of OSK proteins interacting with P7-2. Sequence alignment was performed using BioEdit (Version 7.0.5.3). Conserved amino acids are shaded as per color table. Skp1 domain is shown. Red-asterisks above the aligned sequences shows the 26 key amino acid residues closely related to the interaction between SKP1 and F-box proteins. The eight helixes (H1–H8) found in human SKP1 are indicated by bars.
(TIFF)

S1 Table Primers used in this study. (XLS)
