# Supplementary figures and images for: Rice black streaked dwarf virus P7-2 forms a SCF complex through binding to Oryza sativa SKP1-like proteins, and interacts with GID2 involved in the gibberellin pathway
Source: PLoS One. 2017 May 11;12(5):e0177518. doi: 10.1371/journal.pone.0177518 (PMC5426791; doi:10.1371/journal.pone.0177518)

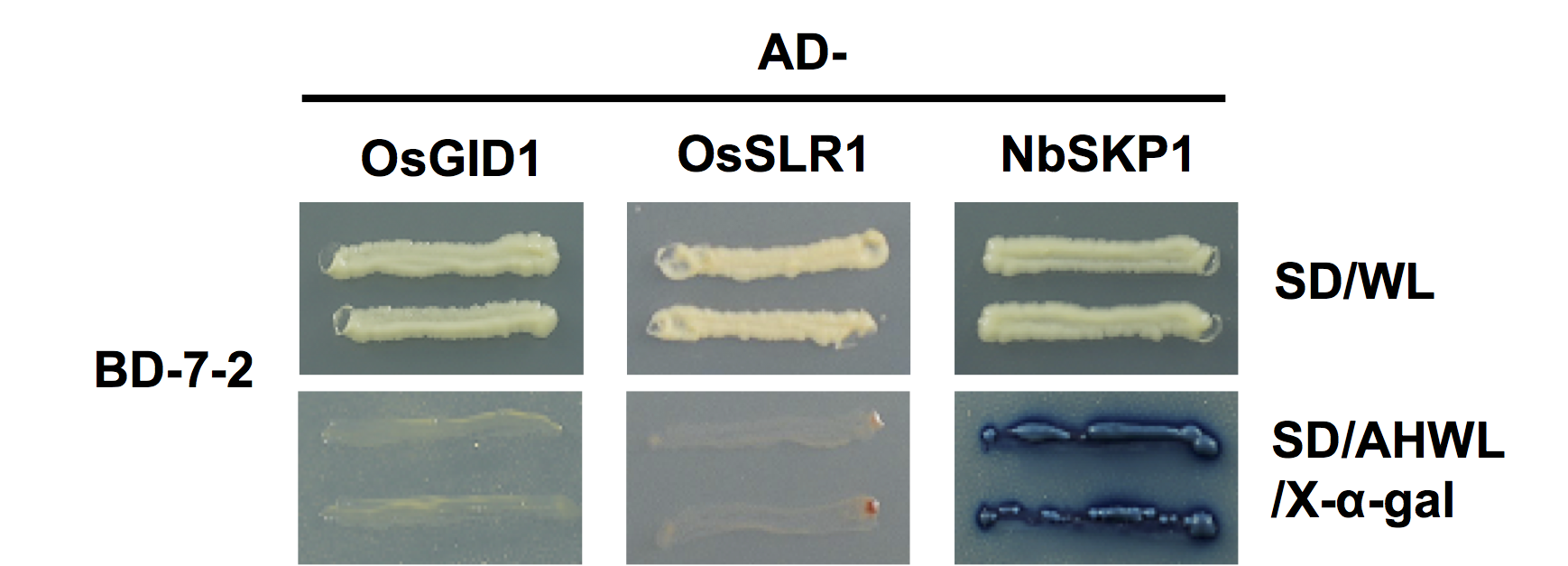

Supplement: S1 Fig — SD/WL was the nonselective medium and SD/AHWL/X-α-gal was the selective medium. SKP1 from Nicotiana benthamiana (NbSKP1) fused to DNA-binding domain was served as positive control. SD/WL, -Trp-Leu; SD/AHWL/X-α-gal, -Ade-His-Trp-Leu containing X-α-gal. (TIFF) [file pone.0177518.s001.tiff]

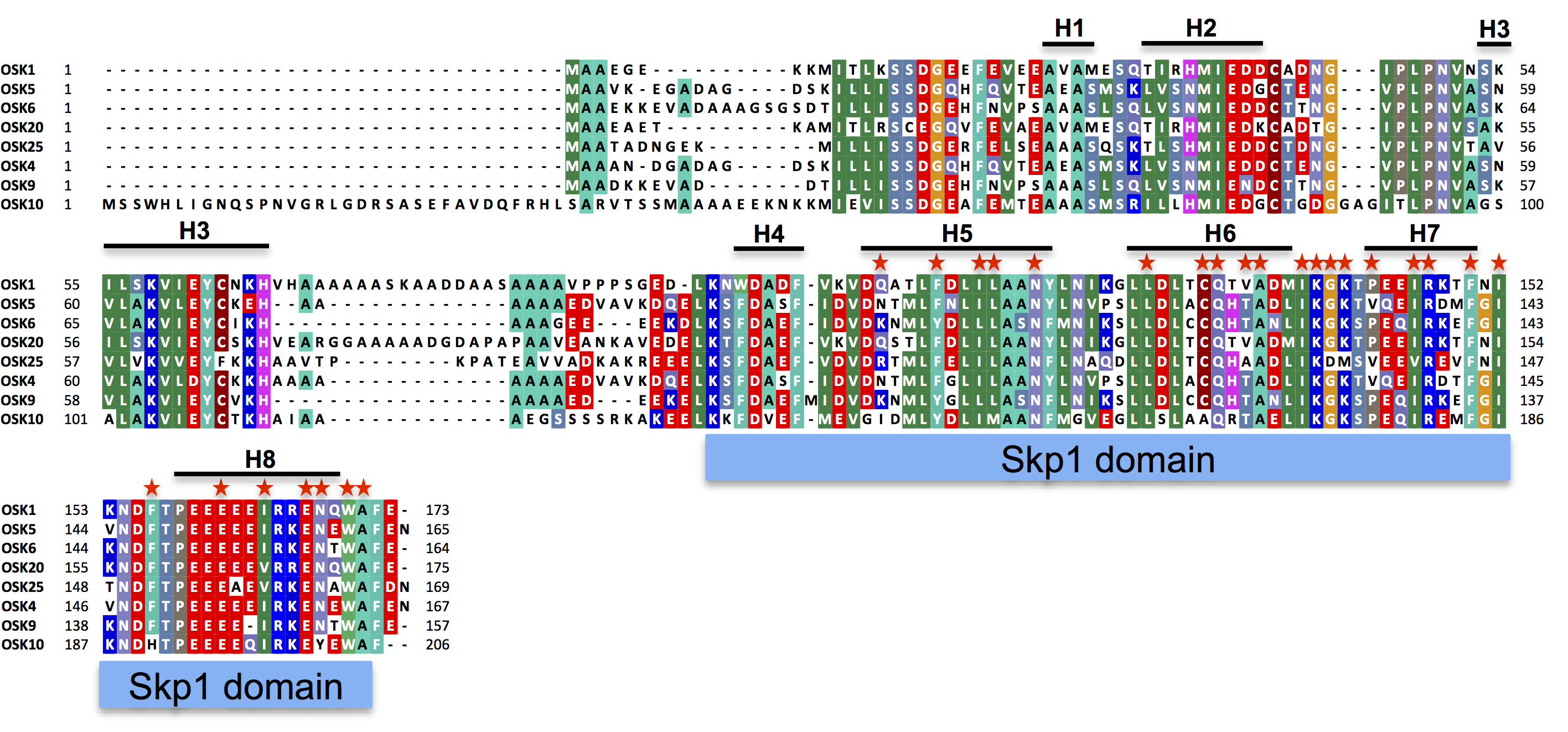

Supplement: S2 Fig — Sequence alignment was performed using BioEdit (Version 7.0.5.3). Conserved amino acids are shaded as per color table. Skp1 domain is shown. Red-asterisks above the aligned sequences shows the 26 key amino acid residues closely related to the interaction between SKP1 and F-box proteins. The eight helixes (H1–H8) found in human SKP1 are indicated by bars. (TIFF) [file pone.0177518.s002.tiff]
